# Green Chemistry and Molecularly Imprinted Membranes

**DOI:** 10.3390/membranes12050472

**Published:** 2022-04-27

**Authors:** Laura Donato, Imen Iben Nasser, Mustapha Majdoub, Enrico Drioli

**Affiliations:** 1 Institute on Membrane Technology, CNR-ITM, University of Calabria, Via P. Bucci, 17/C, 87030 Rende, CS, Italy; e.drioli@itm.cnr.it; 2 Faculté des Sciences de Monastir, Université de Monastir, Bd. de l’Environnement, Monastir 5019, Tunisia; benasserimen@yahoo.fr (I.I.N.); mustaphamajdoub@gmail.com (M.M.); 3 Department of Engineering and of the Environment, University of Calabria, 87030 Rende, CS, Italy; 4 College of Chemical Engineering, Nanjing Tech University, Nanjing 211816, China; 5 Centre of Excellence in Desalination Technology, King Abdulaziz University, Jeddah 21589, Saudi Arabia

**Keywords:** green chemistry, green molecular imprinting, natural materials, molecularly imprinted membranes

## Abstract

Technological progress has made chemistry assume a role of primary importance in our daily life. However, the worsening of the level of environmental pollution is increasingly leading to the realization of more eco-friendly chemical processes due to the advent of green chemistry. The challenge of green chemistry is to produce more and better while consuming and rejecting less. It represents a profitable approach to address environmental problems and the new demands of industrial competitiveness. The concept of green chemistry finds application in several material syntheses such as organic, inorganic, and coordination materials and nanomaterials. One of the different goals pursued in the field of materials science is the application of GC for producing sustainable green polymers and membranes. In this context, extremely relevant is the application of green chemistry in the production of imprinted materials by means of its combination with molecular imprinting technology. Referring to this issue, in the present review, the application of the concept of green chemistry in the production of polymeric materials is discussed. In addition, the principles of green molecular imprinting as well as their application in developing greenificated, imprinted polymers and membranes are presented. In particular, green actions (e.g., the use of harmless chemicals, natural polymers, ultrasound-assisted synthesis and extraction, supercritical CO_2_, etc.) characterizing the imprinting and the post-imprinting process for producing green molecularly imprinted membranes are highlighted.

## 1. Introduction

Chemistry is at the heart of technological progress that stimulates the production of new essentials of modern life. Due to its presence in all areas of our life, such as the materials and the objects that surround us and that we use every day, (food, drugs, fertilizers), chemistry affects all people. In this context, owing to the development and expansion of chemical industries, large amounts of non-degradable chemicals are present in the environment, leading to pollution and toxicity hazard for human, fauna and flora health. Currently, chemistry is strengthened by the emergence of the green chemistry (GC) concept, which is based on producing more and better, but also by bringing the degree of pollution to the lowest level to ensure both environmental protection and health safety. Moreover, green synthesis in chemistry is today one of the essential aspects to be taken into consideration in the development of new products. For achieving these results, GC concept deals with the efficient use of raw materials, the removal of wastes and the avoidance of using toxic and/or hazardous reagents and solvents in the manufacture and application of chemical products [1,2,3,4,5].

As Kharissova et al. reviewed [1], green chemistry finds application in several material syntheses such as organic, inorganic, and coordination materials and nanomaterials. Based on its 12 principles, it is oriented toward many innovative fields of research. Among them, extremely important is the application of green chemistry in the production of imprinted materials by means of its combination with molecular imprinting technology (MIT).

The latter is a powerful advanced strategic approach leading to the production of polymeric materials endowing specific recognition sites able to selectively interact with targeted analytes called “template molecules” [6,7,8,9,10]. These kinds of interactions imitate the molecular recognition mechanisms typical of living systems such as the interactions of receptor–ligand, antigen–antibody and enzyme–substrate, thus conferring to the imprinted materials’ biomimetic features [6,7,11]. Over the last decade, the growing demand for highly selective separation systems has led to a rapid development of molecular imprinting technology due to the high selective recognition, retention and transport properties exhibited by printed materials. Currently, they find a wide variety of application, such as affinity separation, recovery of bioactive compounds and critical raw materials, sensing of substances in clinical and environmental field, water decontamination, and so on [2,7,9,10,11,12,13]. Molecularly imprinted materials are produced in the form of polymers and membranes. Their peculiarity is that they possess specific recognition sites toward a particular compound of interest (called template) and are able to selectively recognize and separate it from complex mixtures containing other analytes, including their structural analogs.

These specific recognition properties render them highly selective and advantageous with respect to their corresponding non-imprinted materials (that are not selective) for achieving specific detection and separation at the molecular level [7,8,9,10,11,12,13,14,15,16,17].

In the case of molecularly imprinted polymers (MIPs), the recognition sites are usually created during the polymerization process. The synthesis of MIPs entails the polymerization of a functional monomer around the template molecules with the aid of a cross-linker. Subsequently, the template is extracted from the neonatal polymer matrix leading to the formation of recognition sites that exhibit high complementarity to it in shape, size and chemical function [8,9,10,11,12,15,16]. Molecularly imprinted membranes (MIMs) represent a special format of imprinted polymers combining their specific recognition properties with the typical features of membrane science.

Membrane processes are advanced and sustainable technologies that are increasingly replacing traditional separation techniques or integrating with them to achieve better utilization of raw materials, greater separation efficiency at lower costs and high value products. This is pursued in the logic of a circular economy, which aims at resource recycling and waste valorization, also considering environmental and human protection as well as economic and social needs. Today, membrane operations and particularly pressure-driven processes are used successfully in various area such as chemical, pharmaceutical, food, biotechnological, water treatment and much more [18,19,20,21,22,23]. However, an increase in selectivity for achieving high separation levels of tailored compounds from complex mixtures is necessary. From this point of view, the creation of specific recognition sites within a membrane matrix (or on its surface) leads to the production of highly selective membranes such as MIMs. The employment of these smart membranes as such or integrated with traditional membranes is promising for developing sustainable green processes.

MIMs are produced via different routes. For example, composite MIMs are prepared via the phase inversion technique embedding pre-synthesized MIP particles within the membrane matrix or copolymerizing a thin layer of an imprinted polymer with the surface of a pre-existing membrane. The phase inversion technique is also applied for creating the recognition sites directly into the membrane matrix during its formation. In this case, template molecules are added to the cast solution, and after membrane formation, their removal frees the membrane’s recognition sites. In this last method, MIPs are not used, and non-composite imprinted membranes are obtained [7,11,12,13,17,18].

MIMs offer several advantages over MIPs. In particular, even if MIPs exhibit high specificity, they suffer of a low loading capacity and a scarce possibility of working in continuous operation mode. However, due to their high crosslinking status, they are poorly processable. Conversely, the exploitation of both the inherent selectivity conferred by the imprinting procedure and the typical features of membrane-based separation processes (continuous mode operation, easy-scale up, large-scale application, mild operating conditions of pressure and temperature, etc.) allow MIMs to overcome these drawbacks and exhibit superior selectivity and separation efficiency [7,9,11,13,17,24,25,26]. Some examples of MIMs application are the detection and separation of biomolecules, the enantiomeric separation, the recovery of bioactive compounds from different matrices, and the removal of pesticides and dyes from water [7,11,13,18,25,26].

It is undeniable that due to its numerous advantages, molecular imprinting technology is attracting more and more attention. However, until now, despite their high separation performance, MIMs are largely used only at the academic level, while their acceptance on the industrial scale is still in an embryonic stage and needs to be heartened. Some factors hindering their application are the reproducibility, the use of high quantities of solvents and reagents often not quite eco-friendly during their fabrication, the increase in membrane cost, and so on. Some of them might be restrained with actions devoted to increasing the visibility and the industrial trials of these innovative tools. In addition, the integration of other membrane operations, such as nanofiltration, ultrafiltration, reverse osmosis, and membrane distillation with MIMs will stimulate their future large-scale application as well as their market. Finally, in this perspective and in view of the strict regulations aimed at the protection of the environment and the reduction of wastes, the scientific community and manufacturers are increasingly exploring the employment of greener strategies in their production processes while maximizing their efficiency and environmental friendliness.

The sustainable potential of molecular imprinting technology has been recently discussed [27]. This review presents an overview of the basic principles and approaches of GC for producing novel green polymers and membranes. Moreover, it discusses the general aspects concerning the application of the GC concept to the production of MIPs. Finally, it highlights the production of green molecularly imprinted membranes in agreement with the principles of green molecular imprinting.

Considering the advantages of GC in many sectors and its relevance in producing eco-friendly high selective separation tools, we are confident that this review will give great contribute to the current research trend in stimulating the production and the employment of molecularly imprinted membranes while applying the concept of green chemistry both on the research and industrial scale.

## 2. Green Chemistry

**Figure 1:**
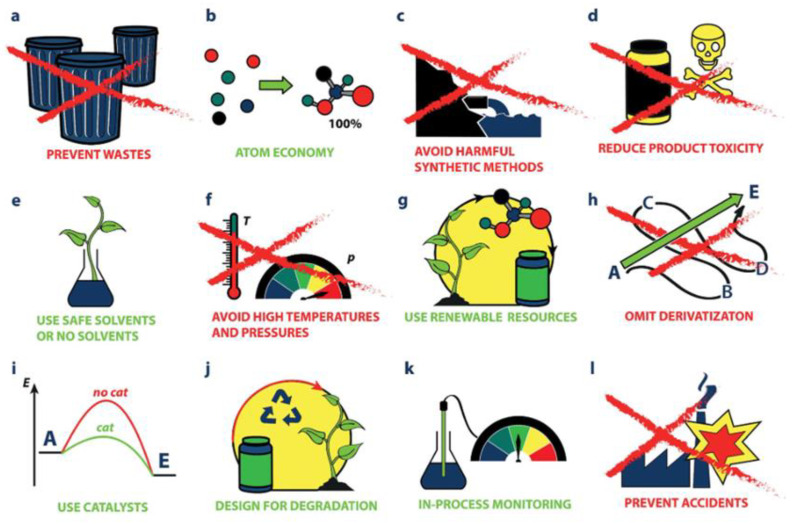
The twelve principles of green chemistry. (Reprinted with permission from Ref [4]. Copyright 2016 John Wiley and Sons).

Sustainable development of syntheses, manufacturing materials and separation processes is becoming more and more a priority on the educational, research and industrial levels for obtaining benefits needed by modern society. In this scenario, chemistry plays a central role in improving the economy, the environment and quality of life. The strict environmental regulations make the chemical industry one of the main sectors affected by environmental problems. These rules require the development of new approaches in chemical processes and in the synthesis of chemical compounds that are safer for the environment and human health. It is in this context that a new branch of chemistry dealing with ecological approaches and called “green chemistry” was born. Today, it attracts great attention and appears as a strategic alternative to traditional chemical processes to reduce the environmental problems and afford the new needs of competitiveness. The concepts of GC is based on the identification and development of sustainable pathways in chemical synthesis and processes, emphasizing environmental and ethical objectives [1,2,3,4]. Green chemistry, also known as “benign chemistry” or “sustainable chemistry”, was born in 1990 in the United States of America, by virtue of initiatives aimed at developing chemical products and processes capable of eliminating or reducing the use and production of hazardous substances. Starting from these good purposes, the “twelve principles of green chemistry” were formulated [1,28]. Figure 1 summarizes them.

As is evident, the principles of GC promote the use of renewable materials and chemical processes with less impact on the environment and with reduction of the quantity of material and risks and dangerous reagents, thus contributing to the sustainability of chemicals and manufacturing processes. According with these principles, many research studies have made significant growth in the green chemistry applications field either at the level of syntheses and reactions or at the level of chemical processes using green routes. For example, the use of GC in chemical reactions requires experimental conditions and synthesis protocols, which include green constituents as solvents, reagents and catalysts [1,2,3,4,28]. From this viewpoint, considering that pharmaceutical and fine chemical industries generate the most abundant wastes, many efforts for reducing them began in the third millennium. For example, for reducing the emission of solvents lost in waste during organic syntheses (as in the production of advanced pharmaceutical intermediates), the use of catalytic methods such as catalysis heterogeneous and bio-catalysis has been foreseen [2,29].

In order to make meaningful comparisons concerning the effectiveness of the various synthetic strategies, their sustainability is evaluated by measuring some parameters such as the environmental factor (E-factor) and atom mass economy (AE). The first indicates the mass of total waste produced versus mass of final obtained product. A value in the range of 25–100 kg indicates a high amount of produced waste with a consequent negative environmental impact [2,30].

The atom economy is a number given from the ratio between the formula weight of the obtained product with respect to the total formula weight of the reactants [2,30]. Assuming exact stoichiometric amounts of starting ingredients and a theoretical chemical yield, it is useful for a quick prediction of the waste that will be generated in the process. Other green metrics, as for example the mass of process water and of used solvents with respect to the mass of the final product, the net mass of materials used, the energy consumed and so on, are discussed in the literature [30,31,32,33].

Briefly, some of the advantages offered by greener processes are: the avoidance of unneeded wastes; the possibility of recycling solvents, catalysts, and other reagents; the development of lower-hazard reactions and small quantity of reactants, thus preventing disasters; low energy consumption, prevention of contamination, better product quality, and so on [1,2,3,4]. Currently, green strategies lead in producing inorganic and organic compounds, nanomaterials, composites, aerogels, quantum dots, etc. Furthermore, green approaches can help in improving some traditional materials such as ceramics, polymers, adsorbents, bioplastics and biocomposites [1,2,3,4].

Among various applications of GC, great advantages derive from the employment of water and other green solvents in chemical reactions such as aqueous catalysis [34,35,36], of supercritical fluids (i.e., supercritical CO_2_) [37,38], ionic liquids (ILs) [39,40,41], deep eutectic solvents (DES) [42,43,44] and fluorous media [45]. Other green approaches are microwave-assisted and ultrasound-assisted processes [46,47,48], hydro/solvo thermal reactions [49,50], magnetic field-assisted synthesis [51], mechanochemistry [52], and UV irradiation [53,54]. All these approaches and the evolution of GC have been accurately reviewed [1,55,56,57,58,59,60,61], highlighting that over the last 20 years, the principles of green chemistry have strengthened the environmental sustainability of chemical processes.

## 3. Toward Green Polymers and Membranes

One of the different goals pursued in the field of materials science is the application of GC for producing sustainable green polymers and membranes [62,63,64,65,66,67,68,69,70,71,72,73,74,75] as well as inorganic–organic hybrid materials of based on a polymeric matrix holding a small amount of inorganic material (such as carbon-based nanotubes, metal nanoparticles and graphene oxide) [76,77,78,79,80,81]. Green chemistry has been applied for fabricating numerous biopolymers, biopolymer-based membranes [64,66] and different synthetic polymers, such as acrylic-based polymers [82], poly(vinyl) chloride [83], polyurethane [84], and so on. Synthetic processes include the use of biomass-based sources [85] and renewable raw monomers such as triglycerides, terpenes, allylic and olefinic monomers [85,86]. An important aspect dealing with the production of hybrid materials is the compatibility of both organic and inorganic ones, and for achieving this goal, surfactants are often used. In this context, microwave irradiation for liquefying lignin as the starting material for the synthesis of flexible polyurethane was performed [87]. A microwave-assisted method for producing piperazine-containing bisphenol formaldehyde polymer was also applied [88].

**Figure 2:**
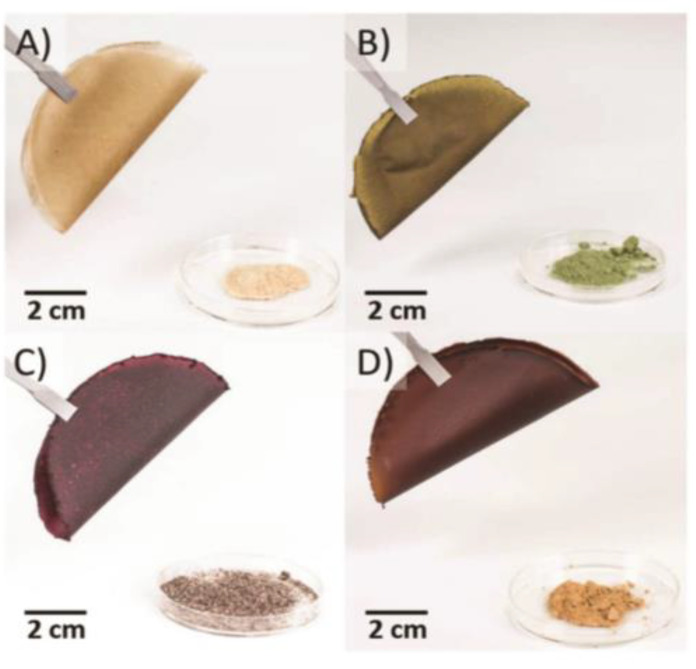
Bioplastic films obtained from vegetable wastes in mild aqueous conditions: (**A**) carrot bioplastic; (**B**) parsley bioplasgtic; (**C**); radicchio bioplastic; (**D**) cauliflower bioplastic. (Reprinted with permission from Ref. [89]. Copyright 2018 Royal Society of Chemistry).

An innovative strategic application of GC takes place in the valorization of vegetable wastes for preparing biodegradable bioplastic films. Perrotto et al. [89] produced environmentally friendly and freestanding bioplastic-based films in a simple one steep process (in aqueous solutions of hydrochloric acid), exploiting different waste matrices (carrot, parsley, radicchio and cauliflower). They maintained the color of starting material and antioxidant properties. Moreover, these films exhibited mechanical properties similar to those of traditional synthetic plastics (i.e., polypropylene, polyethylene, polystyrene, poly (methyl methacrylate) (see Figure 2) [89].

The combination of these bioplastics with other polymeric materials produced composite films with reduced oxygen permeability and increased mechanical resistance, suitable for packaging application. One example is the polyvinyl acetate/carrot bioplastic blend [89]. Other cases of food waste valorization are the synthesis of cellulose-based bioplastics from wastes of parsley and spinach stems, rice hulls, and cocoa pod husks by digestion in trifluoroacetic acid (TFA), casting, and subsequent solvent evaporation [90]. Protein-based polymers and starch-based plastics [91,92] are also produced. The successful recycling of non-edible parts of vegetables demonstrated that it is possible to substitute non-renewable plastic resources with renewable biodegradable resources, thus centering on green chemistry and circular economy concepts [93,94].

**Figure 3:**
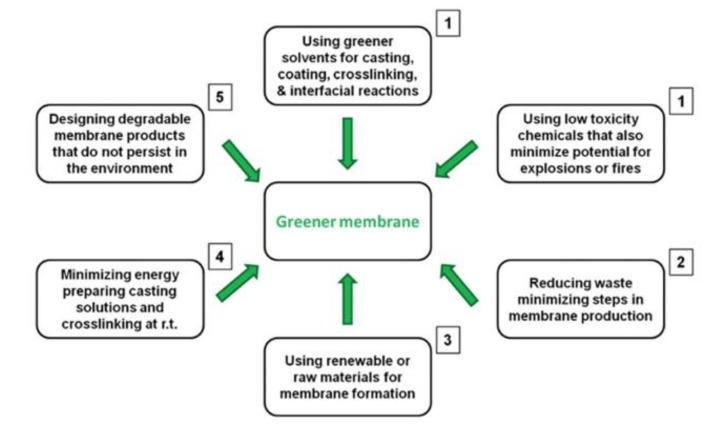
Strategy to develop greener membranes following the principles of green chemistry. The number next to the boxes represent the ranking in order of priority according to their contribution to making a membrane fabrication process greener. (Reprinted with permission from Ref. [95]. Copyright 2014 Royal Society of Chemistry).

The preparation strategies of greener membranes fulfilling the principles of GC were classified by Szekely and co-authors on the basis of the priority of their contributions [95]. Figure 3 summarizes them [95].

**Figure 4:**
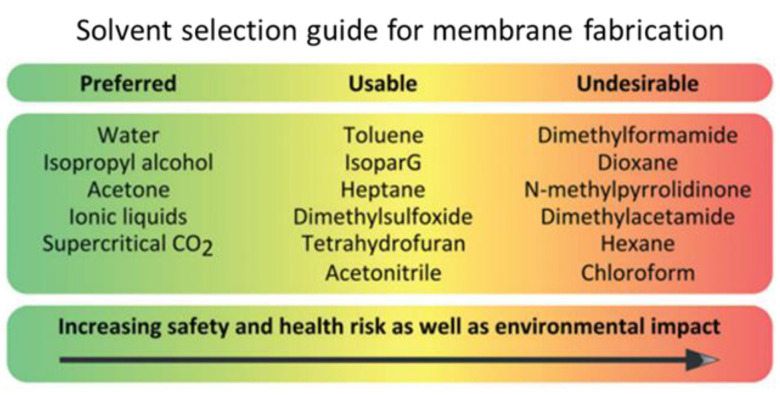
Preferred and undesirable solvents for membrane preparation. (Adapted with permission from Ref. [95]. Copyright 2014 Royal Society of Chemistry).

The first necessities are the use of greener solvents and minimizing the use of toxic chemicals for rendering the process safer. From this viewpoint, the preferred solvents are water, acetone, isopropanol, ionic liquids and supercritical CO_2_, while dimethylformammide, dimethylacetamide, dioxane, hexane, chloroform and N-methylpyrrolidinone are undesired (see Figure 4) [95].

In producing polyimide P84 membranes, Soroko et al. substituted the toxic dimethylformammide and dioxane with the greener acetone and dimethylsulfoxide [96,97]. In addition, the use of inorganic salts (i.e., calcium chloride, sodium tartrate), non-toxic organic molecules (i.e., citric acid) or UV irradiation for post-membrane preparation crosslinking helps in reducing the waste of toxic reagents [97,98,99,100,101]. Another fruitful strategy is the employment of bio-based solvents, such as glycerol and its derivatives, aqueous solutions of carbohydrates and gluconic acid, lignin-derived solvents, fatty acid methyl esters, and so on [102,103]. The second priority deals with the minimization of waste production, energy consumption and operating costs by reducing the steps of the membrane preparation procedure as much as possible. One possible approach is the combination of crosslinking and coagulation in one step, by adding the cross-linker to the cast solution. Third, the use of renewable and degradable materials as bio-based materials is advisable.

The fourth goes for solubilizing and crosslinking polymers at room temperature for reducing energy consumption. The production of degradable membranes is the final greener aspect that aims at the substitution of the conventional petroleum-based membranes with easy degradable bio-based membranes. Nevertheless, the production of bio-based polymers is not yet enough to satisfy the wide request of membrane manufacturing at the industrial level [66,95].

Cellulose, a polysaccharide produced by plants made up of long macromolecular chains of β-D-glucose, is one of the most employed raw materials for preparing bio-based membranes. It is used for preparing polymeric flat-sheet films and hollow-fibers with green synthetic routes as the employment of green solvents such as, methyl lactate, N-methylmorpholine-N-oxide and ionic liquids [66,95,103,104,105,106,107,108]. Cellulose-based polymers are blended with other polymers, or they are hybridized with inorganic materials [109,110,111]. As it was critically reviewed by Galiano et al., bio-based polymeric membranes have also been prepared using chitosan, hyaluronic acid, poly(isoprene), sodium alginate, and more [66].

The global market trend of green chemistry is increasing in different fields, especially in the pharmaceutical sector as well as agro-food processes and packaging, and it was predicted that its values will reach USD 165 billion by the year 2027 [112]. Nevertheless, the production of green separation materials endowed with high specific and selective separation ability at both the ionic and molecular levels, such as imprinted materials, is still in infancy. Therefore, more efforts at the research level, including development and technological transfer, are necessary for assessing the potential of integrating green chemistry with imprinting technology for a possible application of green highly selective tools at a large scale in the near future.

## 4. Green Chemistry in the Synthesis of Molecularly Imprinted Polymers: General Aspects

Imprinting technology is a multidisciplinary approach that bio-mimics the interactions of enzyme–substrate and antigen–antibody occurring in living systems in order to produce selective, resistant and reusable imprinted materials (i.e., polymers and membranes). These advanced separation tools exhibit high selective recognition and separation properties toward a specific compound of interest (both called “template” [7,12,13,113,114,115,116]. Currently, molecularly imprinted polymers are produced at the research and industrial levels and are employed in different areas of science and technology [13,114,116,117,118]. Prominent applications include chromatographic separation [119,120], solid-phase extraction and microsolid-phase extraction [121,122,123], sensing [124,125,126], chiral separation [127], drug delivery [128,129], and so on. This high specificity is due to the presence of recognition sites complementary to the template molecules and capable of recognizing them in a selective way from complex mixtures. The selective recognition sites are created during the synthesis of MIPs and make them advantageous with respect to traditional non-imprinted polymers for obtaining tailored separations at the molecular level [8,9,10,11,12,15,16].

Nevertheless, even if the features of these materials are in line with the GC concept, conventionally used reagents and strategies are not accurately green. Therefore, environmental awareness, the objective of an efficient use of raw materials and the simultaneous increased demand for highly selective separation systems, has led to the application of the principles of green chemistry to their production. This was pursued considering the needs of sustainable practices and GC advances, as well discussed by Erythropel et al., who proposed the “green chemisTREE” as a window display for the actions and continued growth of green chemistry [130].

**Figure 5:**
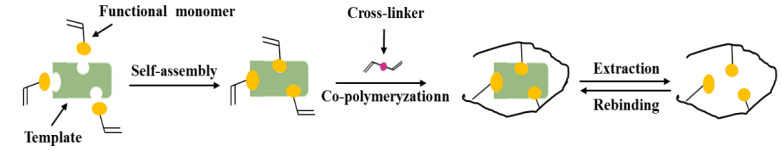
Representation of the MIPs synthetic process.

The traditional synthesis of an imprinted polymer involves the polymerization of a functional monomer in the presence of the template, with the aid of a crosslinker and an initiator. During the process, the functional monomer polymerizes around the template, which remains entrapped in the nascent polymer chains. The removal of the template after polymerization reveals specific recognition sites distributed into the neonatal imprinted polymeric matrix. These sites are complementary to the template in terms of chemical function, shape and size, and are able to recognize and separate it from a complex mixture containing other compounds, including its structural homologues and opposite enantiomers [12,13,113,131]. Figure 5 shows a schematic representation of the synthetic procedure of MIPs.

Relevant aspects of the overall synthetic process include:

The necessary chemical complementarity between the template and the functional monomer, which forms pre-polymerization complexes (via covalent or non-covalent binding);

The choice of a reaction solvent non-interfering with the monomer–template interactions for warranting the formation of efficient recognition sites;

The use of the cross-linker for ensuring the formation of a three-dimensional cross-linked network and for stabilizing the recognition sites;

The employment of an appropriate organic solvent (or other methods) for removing the template from the imprinted matrix and for freeing the recognition sites.

**Figure 6:**
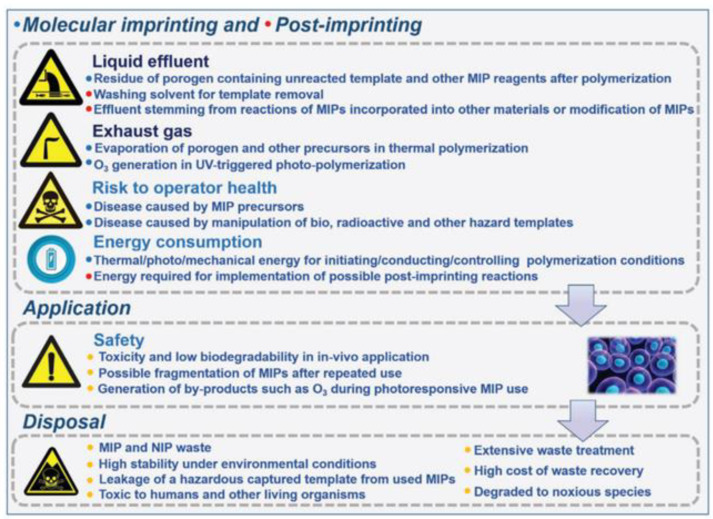
Criticism of unsustainable molecular imprinting technology covering imprinting and post-imprinting application and disposal. (Reprinted with permission from Ref. [132]. Copyright 2021 John Wiley and Sons).

From the above, it is clear how all these aspects of MIPs synthesis have an influence on the environment and on social impact. Furthermore, the health risk of imprinters and the negative influence of the poor degradability of MIPs that end up in the environment after their use should be consider. In a recent review [132], Arabi et al. listed the critical sides of the traditional imprinting technology (see Figure 6).

**Figure 7:**
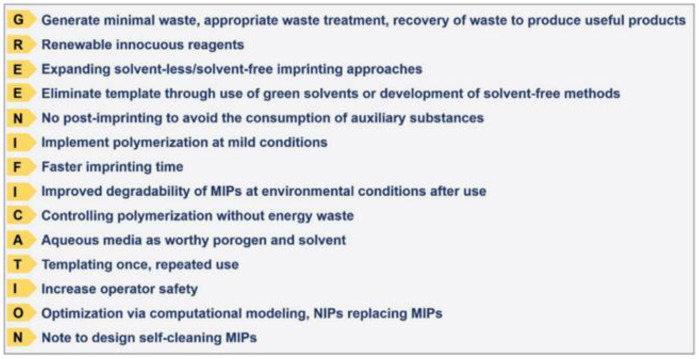
The fourteen principles of green molecular imprinting expressed as the mnemonic device “GREENIFICATION.” (Reprinted with permission from Ref. [132]. Copyright 2021 John Wiley and Sons).

The authors critically evidenced the unsustainable points of the imprinting and post-imprinting steps, including the application and disposal of MIPs. This research group also coined the term “greenification” to present for the first time the fourteen green principles of imprinting technology [132] as a general guide for the development of green MIPs (see Figure 7).

These principles cover different aspects, ranging from the employment of non-toxic (or low-level) reagents and synthetic methods to the fabrication of self-cleaning MIPs in a short time and the optimization with the aid of computational design prioritizing operator safety [132].

For example, traditional largely used functional monomers such as methacrylic acid, acrylic acid, and 4-vynil pyridine, as well as the cross-linkers ethylene glycol dimethacrylate, trimethylolpropane trimethacrylate, and divinylbenzene, are known as toxic chemical compounds, and according to the principles of green imprinting, they begin to be replaced with harmless or environmentally friendly monomers. In this context, room temperature ionic liquids (RTILs) and deep eutectic solvents (DESs) have emerged as green functional monomers and solvents [133,134,135]. Ionic liquids are non-volatile and non-flammable compounds miscible with a wide number of organic solvents. They exhibit low vapor pressure and high boiling point, high stability, ionic conductivity and viscosity. RTILs are able to interact with different organic compounds and bio-macromolecules through the formation of hydrogen bonds, anion-exchange, hydrophobic, electrostaic and π–π interactions. RTIL-base MIP presents excellent recognition properties in an aqueous environment [136,137]. One example is represented by the synthesis of a chlorsulfuron-imprinted MIP using 1-vinyl-3 butyl imidazolium chloride. Binding studies evidenced a higher binding capacity of the MIP (3.88 mg∙g^−1^) toward the template molecules with respect to the non-imprinted polymer (2.96 mg∙g^−1^). Furthermore, in competitive adsorption, this MIP showed a high binding selectivity (47.2%) toward the template with respect to its analogs [138]. In a different work, 1-viny-3-carboxybutyl imidazolium bromide resulted in an efficient functional monomer in the synthesis of a polymer imprinted with synephrine [139]. Table 1 lists some ionic liquids used as functional monomers and their relative template.

Importantly, ionic liquids are not only used as functional monomers and porogens, they are also employed as additives, cross-linkers and dummy templates. These aspects are well discussed in different papers [134,136,159,160,161,162]. However, the employment of RILs is restricted by their high cost. In addition, not all of them are assessed to be non-toxic, and in some cases, it is preferred to use their derivatives [163].

Other strategies involve the use of metal ions [164], boronates [165] and bio-based monomers, etc. [132,166,167]. For example, chitosan, and sodium alginate are natural excellent biocompatible, eco-friendly and cost-effective materials simply polymerizing under mild conditions and interact efficiently with different types of templates (i.e., ions, organic molecules and biomolecules [132,167,168]. Furthermore, inorganic salts such as calcium chloride and N, O-bismethacryloyl ethanolamine (NOBE) resulted a valid alternative to move toward greener crosslinking agents [60,169]. Self-initiated polymerization is also a good strategy for avoiding the use of initiators. For example, it has been demonstrated that acrylic monomers such as 2-hydroxyethyl methacrylate, glycidyl acrylate and methacrylic acid are self-initiating polymerizable functional monomers by a simple excitation to a triplet state [170,171].

Considering that some polymerization methods lead to the production of high volumes of disposal and toxic solvents (e.g., chloroform, dichloromethane, N, N-dimethylformamide, hexane), which can contaminate the environment and operators, are studying new green solvents as alternatives to traditional ones [172]. In this perspective, deep eutectic solvents are emerging as a new generation of green solvents [173]. They consist of two components that are a hydrogen donor (e.g., choline chloride) and hydrogen acceptor (e.g., alcohols, amides, amines, urea). They have similar features to ionic liquids, but they are cheaper, safer, highly biodegradable and can be produced by non-ionic-based compounds [173,174,175,176,177,178]. As it was well discussed in recent review papers [60,133,135], they have an excellent recognition ability in aqueous media and are increasingly employed in imprinting technology. Recently, an imprinted polymer was fabricated using a bio-based deep eutectic solvent for the enrichment of organophosphorous in fruits and vegetables [179]. Adsorption experiments carried out with 5 mL of solution containing 5 mg of MIP and a mix of pesticides having each one an initial concentration of 218 mg∙L^−1^ revealed an excellent adsorption capacity toward all pesticides in a short time (30 s). The highest value (218.62 mg∙g^−1^) was observed in the case of chlorpyrifos, while the adsorption capacity of the non-imprinted polymers was low (48.58 mg∙g^−1^). Another example is the synthesis of MIPs used for the recovery of the bioactive compound synaptic acid from agricultural wastes [180]. More in detail, the imprinted polymer was applied for selectively remove sinapic acid from waste rape seed extract after oil manufacture. The maximum adsorption capacity was 121 mg∙g^−1^, while that of the non-imprinted polymer was 23 mg∙g^−1^. Selectivity studies carried out in the presence of the competing compounds ferulic acid, cinnamic acid, and vanillic acid showed a selectivity factor synaptic acid/competitor of 20.86, 28.77 and 24.26, respectively. Conversely, the non-imprinted polymer was not selective and exhibited similar adsorption capacity toward all tested compounds [180].

Other strategic approaches devoted to the reduction of conventional solvent consumption and emission involve the combination of green porogenic solvents with traditional functional monomers and cross-linkers or solvent reflux during polymerization [181,182]. The supercritical carbon dioxide (CO_2_)-assisted synthesis is also a fruitful alternative to the traditional synthesis employing organic solvents. Supercritical CO_2_, which is obtainable as a high pure subproduct of the industry, combines the properties of gas and liquid states. Owing to its numerous features (apolar, high diffusion coefficient and mass transport capacity, cheap, inert, low viscosity, non-flammable, non-toxic, odorless, recyclable), it represents a sustainable solvent both at the research and industrial levels and is a good porogen of imprinting processes [60,183,184].

Microwave and ultrasound have been present for some years as sustainable strategies used in the imprinting process. More in detail, microwave-assisted synthesis and the ultrasound-assisted synthesis are applied as innovative green approaches during the polymerization step. The use of microwave consistently reduces the polymerization time with respect to traditional heating. This is due to the promotion of high heat transfer into the reaction mixture that facilitated the increase in reaction rate and the decrease in the energy consumption [60,132,185,186]. A reduction of reaction rate is also obtained when employing ultrasound. This is due to the cavitation effect determined by the ultrasonic energy that increases the solubility and the diffusivity of reactants into the polymerization solvent [187,188]. It was demonstrated that MIPs are synthesized with the aid of these green actions, exhibiting similar or higher specific recognition properties of those prepared via traditional routes [189,190,191,192,193,194].

Another extremely important point to consider is the type of chosen template. The use of toxic templates results as hazardous for human health. In some cases, this problem is overcome by the use of non-toxic dummy templates. Bagheri et al. [195] exploited this approach for fabricating dummy molecularly imprinted polymers able to remove acrylamide from biscuit samples. Instead of the toxic acrylamide, the similar propanamide was employed as a dummy template in a synthetic process carried out in a green aqueous environment, thus also avoiding the use of organic solvents [195]. Dummy molecularly imprinted polymers for detecting and quantifying acrylamide in other food matrices have also been synthesized [196]. In a different way, the drug ractopmanine was detected in pig tissues with MIPs synthesized using ritodrine as the dummy template [197]. Another example of a dummy template is the natural isoflavon daidzein in the production of MIPs capable of removing fluoroquinolones from fish samples [198]. Dummy molecularly imprinted resins resulted in efficient solid-phase extraction of plant growth regulators [199].

The template removal from the polymer matrix after both the imprinting process and the subsequent recognition stage is also a crucial issue. First, the traditional process involves the use of large solvent volume. Second, polymer swelling (due to the solvent action) as well as extreme extractive conditions of pH and temperature can alter the created structure of recognition sites, thus negatively affecting their performance. In this scenario, acetic acid and sodium dodecyl sulfate (SDS) are largely used for removing bio-based templates from imprinted polymers even if the surfactant can be adsorbed by the MIP, allowing for the presence of a negative charge [132]. In order to reduce or avoid the volume of the extraction solvent, microwave-assisted extraction, ultrasound-assisted extraction and pressurized hot water extraction were found to be effective in replacing conventional organic solvents [7,132,200]. Lorenzo et al. [201] intensively discussed the mechanism of these strategies adopted for template removal. A good strategy for reducing reactants consumption, waste generation operating cost, and imprinters exposure is multi-template imprinting, which entails the contemporary use of two or more templates aimed at creating their corresponding recognition sites in a unique polymer matrix [202,203,204].

**Figure 8:**
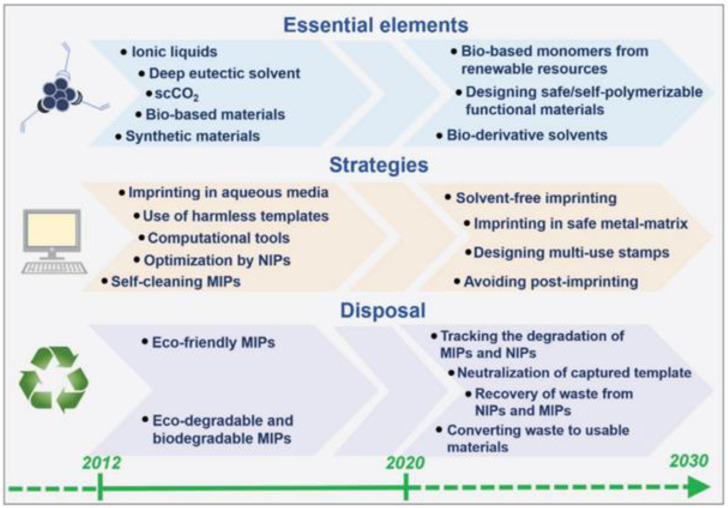
“Greenificated” molecular imprinting technology road map from 2012 to 2030. (Reprinted from ref. [132]. Reprinted with permission from Ref. [132]. Copyright 2021 John Wiley and Sons).

Staying faithful to the principles of greenification, current trends deal with the application of green actions on one or more of the aspects discussed above, also combining eco-friendly and traditionally used chemicals, synthetic routes and post-imprinting phases. Even if greenificated imprinted polymers present the advantages of harmlessness, eco-sustainability and biodegradability, further efforts are still underway to implement production and application. In this context, the roadmap that goes from 2012 to 2030 in Figure 8 envisages the achievement of various objectives [132]. Some of them are the almost total employment of bio-based monomers from renewable resources, solvent-free imprinting, the elimination of the post-imprinting stage, the recovery of wastes from imprinted and their corresponding non-imprinted polymers, and the conversion of wastes in functional materials [132].

In parallel to the research improvement in producing greenificated MIPs, attention was also focalized on the application of the principles of green imprinting technology to the production of green imprinted membranes, which are an advanced form of imprinted polymers, as is discussed in next paragraph.

## 5. Green Molecularly Imprinted Membranes

In accordance with the principles of green molecular imprinting, one of the biggest challenges for scientists is the use of greener approaches and/or materials for the production of green imprinted membranes, which represent a special format of imprinted polymers.

**Figure 9:**
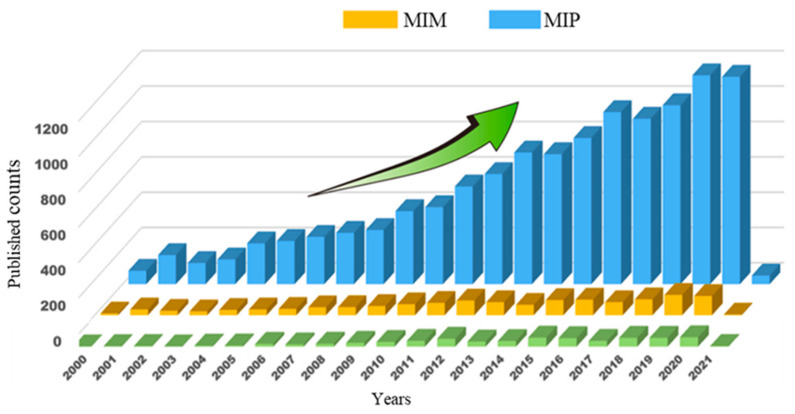
Published papers on MIPs and MIMs from 2000 to 2021 based on web of science core collection. (Reprinted with permission from Ref. [206]. Copyright 2021 Elsevier).

In general, membrane separation processes entail the separation and concentration of one or more desired compounds, employing a membrane as a separation system. The separation occurs owing to the different permeability of the solutes through the membrane under the application of a suitable driving force (i.e., pressure gradient, concentration gradient, etc.). Some typical features rendering competitive membrane-based operations with respect to traditional techniques are easily scaled-up, stability in a wide pH range, work in mild conditions of pressure and temperature, and have no phase change or requirement of additives (or in minimal part), low energy consumption and environmental impact. They can operate as single units or in a continuous integrated manner. The choice of the membrane is an important aspect to consider, because it represents the key to the separation process. Most relevant parameters determining the separation performance are membrane permeability, selectivity and stability [18,19,20,21,22,23]. From this viewpoint, the idea of introducing tailored specificity into a traditional membrane has opened up new frontiers in the field of membrane operations from microscale to nanoscale applications. This is because imprinted membranes are intelligent tools exploiting contemporary typical features of imprinting and membrane technologies, thus offering several advantages with respect to imprinted polymers and traditional membranes [205,206,207,208]. For example, MIPs suffer from a low load capacity and a poor possibility of working continuously, and due to their high level of crosslinking, they are poorly processable. Conversely, MIMs are able to operate in a continuous mode, can be applied at large-scale and show higher binding capacity and separation efficiency. In addition, they exhibit improved selectivity with respect to the traditional ones but preserve their stability and permeability properties and separate structural homologues and enantiomers between them [206,207,208,209]. Molecularly imprinted membranes are applied in different areas both as an alternative to the traditional separation technologies or integrated with them as well as with non-imprinted membranes for obtaining a high purification level of specific molecules in a sustainable way [7,206,210,211,212,213]. However, despite their many advantages and although their production has grown a bit more in recent years, the number of works dealing with the production of MIMs is still low compared to that of MIPs (see Figure 9).

**Figure 10:**
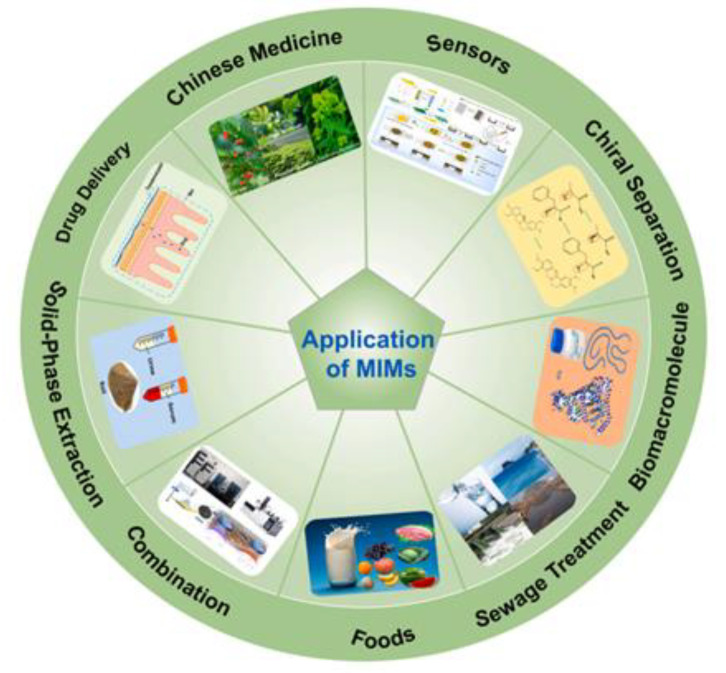
Application areas of MIMs. (Reprinted with permission from Ref. [206]. Copyright 2021 Elsevier).

Therefore, more efforts are necessary for their wide-ranging development, not only in the field of research but also at an industrial level. As summarized in Figure 10, some examples of MIMs application are the separation of macromolecules and drugs and the selective recovery of drugs, bioactive compounds, and herbal ingredients from different matrices [206,213,214,215,216,217,218,219]. Other applications are the clinical monitoring of drugs and toxic compounds [220,221], the drug delivery [222,223,224,225,226], the enantiomeric separation [227,228,229], as well as the detection and removal of contaminants from water and other sources [206,209,230,231,232]. Among all these applications, one example is the production of artemisinin-imprinted composite membranes for the selective separation and purification of the anti-malaria drug artemisinin from ethanol solutions containing its structural homologue artemether. In adsorption experiments, the maximum adsorption capacity of MIMs was 158.85 mg∙g^−1^, while that of the corresponding non-imprinted membrane was 37.35 mg∙g^−1^. Furthermore, the imprinted membrane exhibited an adsorption selectivity for artemisinin/artemether of 2.04. In competitive permeation experiments, the permeate flux of artemisinin was 12.5 mg∙cm^−2^ s^−1^ × 10^−4^, while that of artemether was 2.68 mg∙cm^−2^ s^−1^ × 10^−4^ [216]. Another example is the extraction of the herbal active ingredient Ebracteolata B, which exhibits different pharmacological effects such as anti-tubercle and anti-cancer activities, from *Euphorbia fischeriana* extract [217].

The separation of template molecules is achieved via either their selective retention or facilitated permeation [7,206,209,229,230,233,234].

Molecularly imprinted membranes are prepared in various configurations (flat sheets, hollow fibers, nanofibers) by exploiting different strategies, where the interactions of template-functional monomers and template-recognition sites of the membrane matrix occur via covalent or non-covalent bond (similar to MIPs) [7,13,206,209,213,235,236].

One of the traditional preparation methods of MIMs is the surface imprinting of a pre-existing membrane via the copolymerization of a thin imprinted polymer layer with the surface of a pre-existing membrane (e.g., commercial or previously prepared). This route obtains composite flat-sheets, hollow fibers as well as nanofiber membranes. The application of the phase inversion technique leads to the production of composite MIMs by means of the hybridization of previously synthesized MIP particles with a polymer commonly used for preparing membranes, either via “wet” or “dry” phase inversion (or their combination). These last methods are also useful for preparing non-composite MIMS MIMs using a polymer ad hoc functionalized with chemical functions able to interact with the template molecules. Therefore, in this case, the simultaneous formation of the membrane structure and of its selective recognition sites occur [7,13,206,209,213,229,230,235,236]. As an example of membrane preparation, Donato et al. [229] developed S-naproxen-imprinted membranes via photo-copolymerization of the functional monomer 4-vinylpiridine with the surface of a commercial polypropylene microfiltration membrane. The enantioselective-imprinted membrane exhibited a facilitated permeation of the enantiomer template. At optimized operating conditions (T = 25 °C, pH = 3.4, P = 0.4 bar, ((R,S)-Nap) = 7.0 µg/mL^−1^), the permselectivity factor S-naproxen/R-naproxen was 1.8. The water permeation flux was typical of ultrafiltration. On the contrary, the pristine commercial membrane and the blank non-imprinted membranes were not selective. In particular, the commercial membrane allowed for the permeation of both enantiomers, while the blank membrane exhibited a low permeation rate owing to the absence of the S-naproxen recognition sites [229]. Composite MIMs prepared via the phase inversion technique hybridizing the poly (vinylidene) fluoride matrix with polymer particles imprinted with 4,4-methylendianiline exhibited high specific retention toward the template with respect to non-imprinted membranes and those prepared with the only commercial poly (vinylidene) fluoride [237]. In permeation experiments performed in isopropanol at pressure of 0.1 bar with an initial feed concentration of 10 mg∙L^−1^ (in 100 mL), MIMs containing 33 wt.% of MIP particles showed the highest binding capacity (7.5 µmol∙g^−1^). At the same conditions, the corresponding non-imprinted membrane and the simple PVDF membrane exhibited a binding capacity of 4.4 and 2.0 µmol∙g^−1^, respectively. The permeability of the poly (vinylidene) fluoride-based membrane was in the nanofiltration range, while that of MIM and non-imprinted membranes was typical of ultrafiltration, indicating that the addition of polymer particles to the poly (vinylidene) fluoride matrix increased its permeability performance. Moreover, the MIM exhibited a selectivity factor of 1.82 toward 4,4-ethylendianiline [237].

Over time, these approaches were improved and strategically combined for producing advanced membranes [238,239,240,241,242,243,244,245,246,247,248].

**Figure 11:**
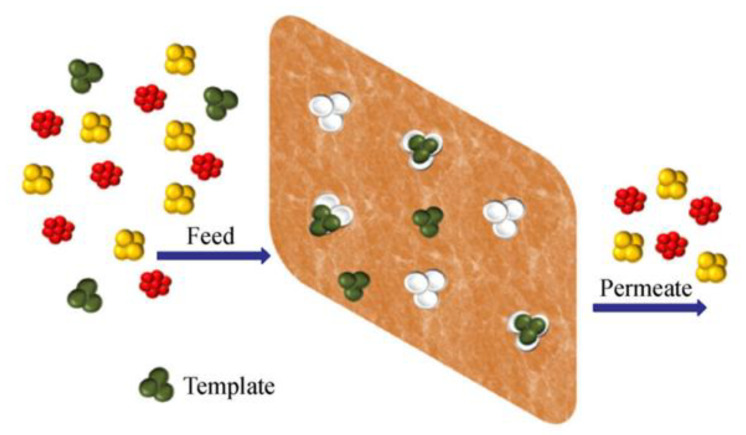
Representation of a flat-sheet molecularly imprinted membrane able to selectively bind the template molecules. (Reprinted with permission from Ref. [7]. Copyright 2021 Springer Nature).

Figure 11 shows the representation of flat-sheet membranes exhibiting selective binding toward template molecules, thus separating them from competing compounds that accumulate in the permeate stream [7].

The high performance of selective separation of MIMs, combined with the typical characteristics of membrane processes, makes their use rational with the principles of green imprinting technology, even if some characteristics relating to their preparation need to be better addressed with new and greener interventions. Similar to the case of MIPs, this is already occurring in part, as for example via the use of more environmentally friendly functional monomers, solvents and polymers that form the membrane structure. Other ecological actions are the application of new synthetic routes or the dummy template and multi-template imprinting, as well as the template extraction with ultrasound, microwave or supercritical CO_2_.

For example, the phase inversion technique is useful for preparing MIMs with safe co-polymers such as poly(acrylonitrile-co-acrylic acid) and natural polymers such as chitosan, sodium alginate, cellulose, β-cyclodextrin-based, etc. In particular, natural polymers (and their derivatives) have emerged as high promising materials that form membranes owing to their low cost, eco-friendly features as well as the abundance of active chemical functions (i.e., amino, carboxyl and hydroxyl, groups) with affinity toward many compounds and establishing with them multiple interactions [249,250,251,252]. An important aspect of the employment of these materials is the possibility of avoiding in some cases the polymerization process, while leading the formation of a “polymer–template complex”. This is due to the interactions between the functional groups of the template and the complementary chemical moieties of the polymeric material that form the membrane [250,251,253,254]. This structure is stabilized with the aid of a cross-linker both during or after membrane formation. These types of polymer–template interactions comprise hydrogen bonding, electrostatic and π–π interactions, and van der Waals forces. A problem with these materials is the structural stability in severe conditions; thus, the scientific community is making efforts in the direction of producing more stable innovative bio-based MIMs. Table 2 reports some examples on natural materials used in fabricating innovative MIMs.

**Figure 12:**
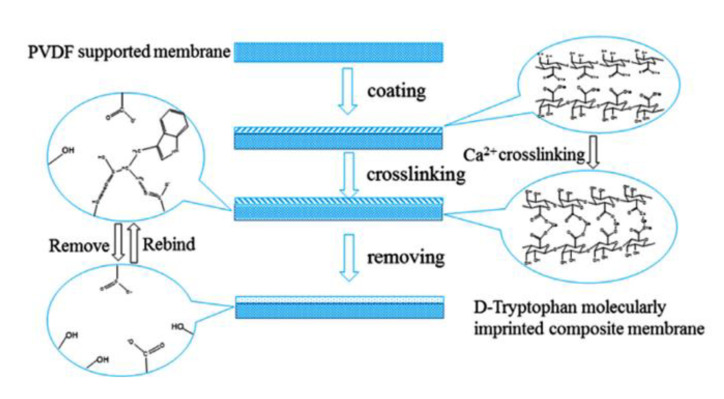
Illustration of the preparation process of composite d-tryptophan-sodium alginate/ poly (vinylidene) fluoride imprinted membrane. (Reprinted with permission from Ref. [254]. Copyright 2017 Elsevier).

In developing green membranes, natural polymers are used as such for direct membrane preparation or as membrane support, coatings or additives for preparing composite membranes via the simultaneous use of traditional polymers (e.g., poly (vinylidene) fluoride, polysulfone, poly (ether sulfone) polyacrilonitrile, etc.). Among natural materials, chitosan possesses both amino and hydroxyl groups, the presence of hydroxyl groups characterizes cellulose, and sodium alginate is a polyelectrolyte rich in carboxyl groups. The macrocyclic β-cyclodextrin consists of an external hydrophilic part and a hydrophobic inner cavity playing a key role in the recognition process. Owing to the biocompatibility, biodegradability and non-toxicity of natural polymers, MIMs prepared with them are suitable for application in the medical, nutraceutical and pharmaceutical fields as well as in water treatment. For example, bacterial cellulose was used for producing both diosgenin-imprinted membranes [250] and quercetin-imprinted [251] membranes, which exhibited a sustained selective release of the template molecules. Recognition sites were created directly into the polymer matrix during the membrane formation step via the phase inversion technique. In a different way, chitosan was used for fabricating a MIM-based sensor able to selectively detect and remove the 4-nitrophenol from drinking water [260]. In batch adsorption studies, this membrane exhibited a maximum adsorption of 723.25 μmol∙g^−1^ of 4-nitrophenol, while the adsorption capacity exhibited by the corresponding non-imprinted membrane was 517.69 μmol∙g^−1^. In addition, MIMs were selective with respect to competing phenol, 3-nitrophenol and 4-methoxyphenol, while non-imprinted membranes were not selective. The treatment of real samples (containing 7.19 μmol∙L^−1^ of this toxic phenolic compound) with MIMs leads to a removal efficiency of 70.6% [260]. More recently, sodium alginate resulted in high efficiency as a polymer, forming enantioselective MIMs with tailored recognition sites specific for d-tryptophan [266]. After their formation, the membranes were crosslinked with calcium chloride by the coordination of two carboxyl groups of the natural polymer and one Ca^2+^ ion exchanged with Na^+^. In pressure-driven permeation tests, these innovative smart membranes were able to separate tryptophan isomers from a racemic solution thorough a facilitated permeation of the template enantiomer. At an operating transmembrane pressure of 0.2 MPa, feed concentration of 0.5 mmol∙L^−1^ and pH above the isoelectric point of tryptophan (5.89), the permeation flux of d-tryptophan was 5.8 × 10^−5^ mol∙m^−2^∙h^−1^, and the permeation enantiomeric excess was about 99% (membrane thickness was 0.02 mm) [266]. Conversely, the non-imprinted membrane showed a permeation flux of almost two times lower (2.91 × 10^−5^ mol∙m^−2^∙h^−1^). Composite MIMs were also prepared via coating the surface of a poly (vinylidene) fluoride membrane with a d-tryptophan-imprinted sodium alginate film, as is shown in Figure 12 [254].

After the coating step and before the template extraction, the new composite MIMs were crosslinked with calcium chloride. Optimized membranes with an imprinted layer thickness of 0.02 mm exhibited a water flux of 6.46 L∙m^−2^∙h^−1^, a d-tryptophan flux of about 1.3 L∙m^−2^∙h^−1^ and an enantiomeric excess of 99.13 [254]. Electrospun methylene blue-sodium alginate/polyethylene oxide imprinted nanofiber membranes [267] and methyl orange-TiO_2_/calcium alginate hydrogel as a matrix [268] were also produced. The binding capacity of these membranes toward the template dye was 14.13 and 3186.7 mg∙g^−1^, respectively. Recently, for producing green tetracycline-imprinted nanocomposite membranes, a biomass-based strategy was developed [273]. In this context, biomass-activated imprinted carbon nanoparticles were embedded into the matrix of porous cellulose acetate/chitosan-blended membranes via phase inversion [273]. These obtained hybrid imprinted membranes exhibited a high permeate rate of the template molecules with respect to the competing structural analog oxytetracycline. The permselectivity factor was 2.4. Other examples of biomaterials are lignin, starch and water-soluble proteins [253,274]. For increasing the number of recognition sites, these materials are also functionalized via esterification, etherification, graft copolymerization, oxidation and Schiff’s base reaction, thus producing biopolymers derivatives [253].

**Figure 13:**
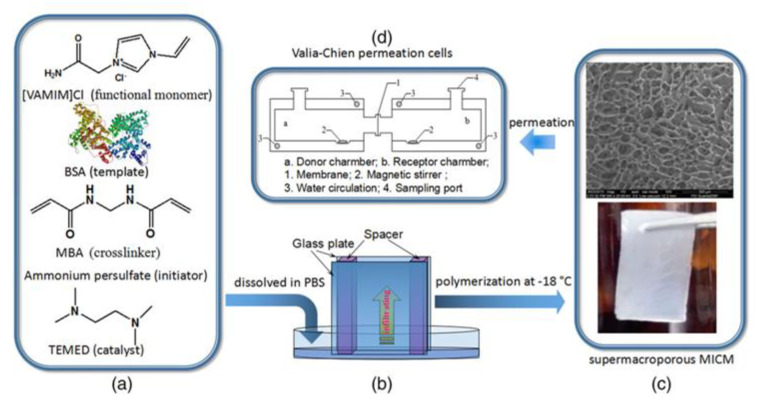
Pre-polymerization mixture components during the synthesis of the cryogel bovine serum albumin-imprinted membrane fabricated by Fan et al. (**a**); infiltration of the mixture between two glass plates (**b**); SEM image (upper) and photo (down) of the obtained free-standing flat-sheet membrane (**c**); diffusion cell used in permeation tests (**d**). (Reprinted with permission from Ref. [278]. Copyright 2018 John Wiley and Sons).

In agreement with the principles of the green molecular imprinting, the employment of greener solvents such as acetone, dimethylsulfoxide, ethanol, isopropanol, ionic liquids, water and deep eutectic solvents for membrane preparation is also continuously increasing as an alternative to the traditional toxic solvents (e.g., dimethylacetammide and dimethylformammide and chloroform). In addition, multi-template imprinting, the use of less toxic functional monomers, initiators and cross-linkers, and more green template extraction strategies is growing [27,275]. A thin polymeric layer imprinted with thymopentin was polymerized on the surface of a regenerated cellulose acetate membrane using the ionic liquid 1-vinyl-3-ethyl acetate imidazolium chloride as a functional monomer [276]. Membranes were tested in solid-phase extraction disks for purifying thymopentin. Scatchard analysis indicated the presence of both high and low affinity binding sites exhibiting a maximum binding capacity equal to 13.07 and 8.04 mg∙g^−1^, respectively [276]. In another case, the ionic liquid 1-butyl-3-methylimidazolium chloride was used as a co-additive for preparing blended salicylic acid-imprinted cellulose acetate/polyethylene glycol-4000 membranes. The aim was the recovery of this active pharmaceutical ingredient from wastewaters [277]. Membranes exhibited higher binding affinity with respect to those prepared without the ionic liquid. In addition, the selectivity factor for salicylic acid with respect to the competing compounds p-hydroxybenzoic acid and phenol was 5.85 and 5.90, respectively [278]. Fan and coauthors developed a macroporous cryogel-imprinted membrane using (1-vinyl-3-(2-amino-2-oxoethyl) imidazolium chloride as a functional monomer and bovine serum albumin as a model template protein [279], in a phosphate buffer as solvent and porogen. The pre-polymerization mixture was infiltrated between two glass plates and the subsequent polymerization took place at −18 °C, leading to the formation of a macroporous membrane structure having a uniform distribution of pores (see Figure 13).

Permeation tests carried out in a diffusion cell with solutions containing the template and the similar human serum albumin (having each one the concentration of 0.4 mg∙L^−1^) evidenced a high transport rate of the template molecules: the permeation amount of bovine serum albumin was 2.82 mg∙cm^−2^, while that of the competing protein was 1.63 mg∙cm^−2^ [278].

Other syntheses, involving the application of the dummy template strategy, led to the production of innovative MIMs. For example, the separation of the herbal Chinese medicine (anti-malaria) artemisinin from the contaminant artemether was achieved with composite membranes imprinted with the dummy compound artesunate. This was because artemisinin has no chemical groups that can interact with functional monomers [279]. Membrane were prepared via the phase inversion adding pre-synthesized artesunate–MIP particles to a poly (vinylidene) fluoride cast solution.

**Figure 14:**
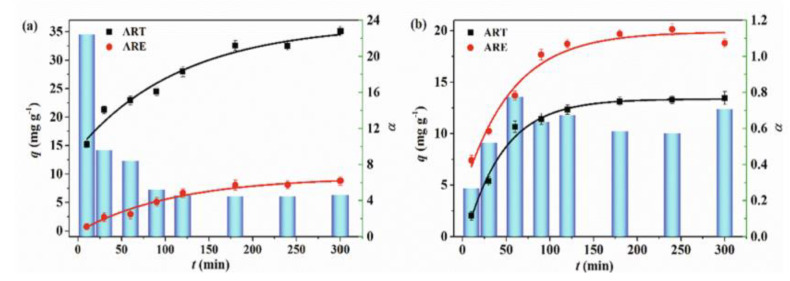
Adsorption capacity of poly (vinylidene) fluoride artesunate-imprinted (**a**) and non-imprinted membrane (**b**) (containing the 16.6 wt.% of MIP particles) toward artemisinin and artemether as well as selectivity factor (α) in time. Artesunate was used as a dummy template. Concentration of each analyte in the feed solution = 200 mg∙L^−1^; transmembrane pressure = 0.1 MPa; temperature = 25 °C; flow rate = 17 mL∙min^−1^; active membrane area = 21.23 cm^2^. (Reprinted with permission from Ref. [279]. Copyright 2021 Elsevier).

Figure 14 shows the behavior of the adsorption capacity and the selectivity factor (α) artemisinin/artemether of the best membrane.

In another work, the detection of the mycotoxin citrinin in rice was accomplished using membranes imprinted with the less toxic dummy template 1-napthol [280]. Recently, in an eco-friendly synthesis involving the use of 1-vinylimidazole as a functional monomer and mild photo-co-polymerization conditions on the surface of a nylon-66 membrane, gatifloxacin was used as a dummy template. The prepared composite MIMs allowed for the simultaneous recognition and extraction of the antibiotics enrofloxacin and ciprofloxacin from egg samples [281].

The chemical structure of some investigated compounds and that of their relative dummy templates used in MIMs fabrication are reported in Table 3.

In a different approach, cinnamic and ferulic acids were used simultaneously for fabricating dual-template imprinted membranes exhibiting permeability typical of ultrafiltration and capable of detecting them in cereal samples [284].

In addition to the aspect of template use and extraction discussed in the previous paragraph, (limiting the use of toxic or precious templates as well as solvent consumption for their removal), the dummy template and the multi-template imprinting strategies are useful to control/avoid the template bleeding that sometimes represents a drawback of the subsequent recognition process. This is because possible template traces remaining in the membrane can negatively affect precise analytical determinations if released [176,285,286]. A contribution in this direction also comes from the use of supercritical CO_2_ both during the polymerization step and during template extraction [287,288,289].

As is evident, the combination of the concept of green chemistry with that of molecular imprinting technology has proven successful in the development of “green intelligent membranes”, which are promising for application in different sectors characterizing our life. However, it is necessary to make further efforts to use them now more than before on a large scale and on the industrial level, even in new integrated processes that require high selective separation efficiency.

## 6. Conclusions

Over time, human necessities and technological progress have allowed for contemporary increases in chemical processes at the research and industrial levels. However, the consumption of large solvent, toxicity of some used materials, and disposal problems have led to a status quo no longer sustainable both from the points of view of human health and from that of the host planet. The development of more eco-friendly processes has therefore become an emergency, allowing for the advent of green chemistry. According to its 12 principles based on ecological approaches, it plays a key role as a strategic alternative to the traditional chemical processes for reducing environmental problems and coping with new requirements of sustainability and economic affordability. Today, green chemistry finds application in organic and inorganic syntheses, in chemical reactions, and separation processes, as well as in the production of greener polymers and membranes, such as biopolymers and biopolymer-based membranes via the valorization of wastes. In this scenario, the world of molecular imprinting has embraced the concept of green chemistry and the current trend is devoted to the development of eco-friendly processes for producing green molecularly imprinted materials.

In particular, in agreement with the principles of green molecular imprinting and with their high selective separation performance, green MIMs are promising efficient tools for application in different areas. Strategic green actions characterizing their production are focalized on the minimization of waste production and energy and solvents consumption as well as on the use of harmless chemicals. They are realized with the aid of computational design, prioritizing operator security and including the use of greener or natural polymers as membrane-forming material, greener functional monomers, cross-linkers and solvents such as ionic liquids, deep eutectic solvents, acetone, dimethylsulfoxide, ethanol, water and supercritical CO_2_. The dummy template imprinting and the multi-template imprinting strategies represent other effective approaches that are also effective in limiting the use of toxic or precious templates and in avoiding the template bleeding problem.

The typical characteristics of membrane processes have allowed them to spread widely with great success, predominantly in the case of pressure-driven membrane operations. Conversely, despite the excellent properties of molecularly imprinted membranes, there is still considerable work to accomplish for better exploitation of the combination of green chemistry with imprinting technology for their possible application at a large scale in the near future. From this viewpoint, and taking present the greenificated roadmap from 2012 to 2030 of green imprinting technology, this review represents an opportunity for stimulating the awareness of exploring other green aspects of MIMs production for enhancing their sustainability and environmental remediation. In this perspective, it is legitimate to predict that the production of advanced green imprinted membranes and their integration with traditional membrane operations such as ultrafiltration, nanofiltration, reverse osmosis or membrane distillation will make it possible to market them.

## Figures and Tables

**Table 1:** Some examples of ionic liquids used as functional monomers in the synthesis of imprinted polymers.

Functional Monomer	Solvent	Template	Ref.
1-Allyl-3-ethylimidazolium bromide ([AEIM]Br)	Water	Phenylephrine (dummy template of clenbuterol)	[140]
1-allyl-3- ethylimidazolium hexafluorophosphate;	Water and chloroform	4,4–Dichlorobenzhydrol	[141]
3-(anthracen-9-ylmethyl)-1-vinyl1H-imidazol-3-ium chloride;	Methanol	p-Nitroaniline	[142]
1-[3-(N-cystamine)propyl]-3-vinylimidazolium tetrafluoroborate;	Water	a-Fetoprotein	[143]
1-Ethyl- 3-methylimidazolium tetrafluoroborate ([EMIM][BF4]),	ethanol/water	Patulin	[144]
1-(a-methyl acrylate)-3-methylimidazolium bromide;	Methanol and water	Caffeine	[145]
1-vinyl-3-methylimidazolium chloride	Acetonitrile and water	Benzoic acid (dummy template of salicylic acid)	[146]
1-allyl-3-methylimidazolium bromide	Acetonitrile	Bromide (Z)-3-(chloromethylene)-6-flourothiochroman-4-one	[147]
1-allyl-3-vinylimidazolium chloride	Water and ethanol	Imiquimod	[148]
1-allyl-3-vinylimidazolium chloride;	methanol	Sulfamonomethoxine	[149]
1-(Triethoxysilyl) propyl-3aminopropylimidazole bromide	Tetrahydrofuran and methanol	Bisphenol A (dummy template of organochlorines)	[150]
1-vinyl-3 butyl imidazolium chloride	Water	Lysozyme	[151]
1-Vinyl-3-ethylimidazolium bromide	Water	Ochratoxin A	[152]
1-Viny-3-carboxybutyl imidazolium bromide	Methanol and water	Synephrine	[139]
1-vinyl-3 butyl imidazolium tetrafluoroborate	Methanol	Cyhalothrin	[153]
1-vinyl-3-propylimidazole sulfonate	Water	Hemoglobin	[154]
1,6-hexa-3,30 -bis-1-vinylimidazolium bromine	Water	Levofloxacin	[155]
3-(3-aminopropyl)-1vinylimidazolium chloride	Water	Bovine serum albumin	[156]
Mono-6A-deoxy-6-(1-vinyl imidazolium)-β-cyclodextrin tosylate	Phosphate buffer	C terminal peptides of cytochrome C	[157]
3-Propyl-1-vinyl imidazolium bromide	Methanol and water	Amoxicillin	[158]

**Table 2:** Some examples of natural materials used in the production of MIMs.

Natural Material	Template	Application	Ref.
Cellulose 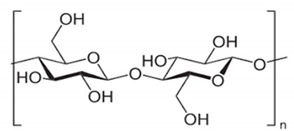	Diosgenin	Sustained release and selective separation	[250]
	Gentamicin	Controlled delivery	[255]
	Myoglobin	Sensing in biological media	[256]
	Quercetin	Sustained release	[223]
	Vanilline	Selective separation	[257]
Chitosan 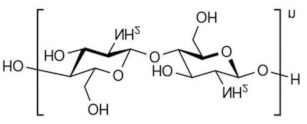	Chlorogenic acid	Selective separation	[258]
	L-Mandelic acid	Enantioseparation	[259]
	4-nitrophenol	Water treatment	[260]
	Naringin	Debittering	[261]
	L-Phenylalanine	Enantioseparation	[262]
	L-Tryptophan	Enantioseparation	[263]
	L-tyrosine	Selective separation	[264]
Sodium alginate 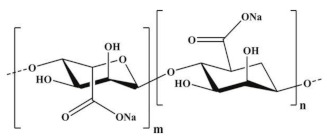	Bovine serum albumin	Adsorption and electrochemical detection in aqueous phase	[265]
	D-Tryptophan	Enantioseparation	[266]
	Methylene blue	Removal from water	[267]
	Methyl orange	Removal from water	[268]
β-cyclodextrin 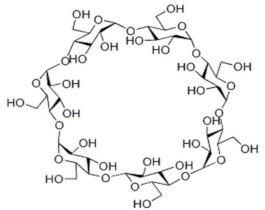	Bisphenol A	Sensing in water	[269]
	Butyl benzyl phthalate and dibutyl phthalate (dual templates)	Sensing in water	[270]
	Ciprofloxacin	Selective separation	[271]
	Triclosan and polychlorophenols	Sensing in water	[272]

**Table 3:** Some target molecules and their relative dummy template used in green MIMs production.

Target Compound	Dummy Template	Application	Ref.
Artemisinin 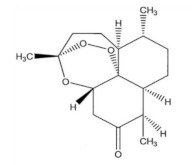	Artesunate 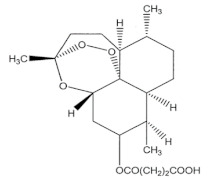	Separation from similar artemether	[279]
Citrinin 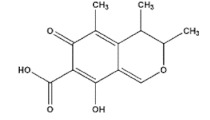	1-Napthol 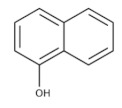	Detection in rice	[280]
Enrofloxacin 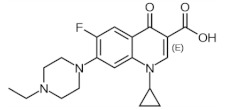	Gatfloxacin 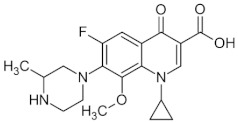	Detection and removal from eggs	[281]
Lovastatin 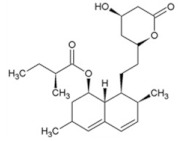	Lovastatin acid 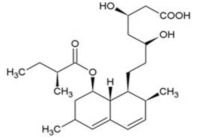	Separation of statins	[282]
Vardefanil 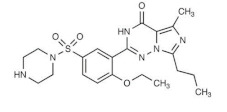	Sildefanil 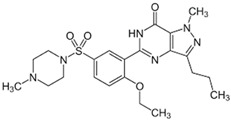	Solid-phase extraction	[283]
Zearalenone 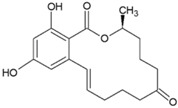	Cyclododecyl-2,4-dihydroxybenzoate 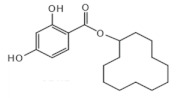	Detection in cereal samples for inspecting *fusarium* contamination	[284]
